# Effects of Nitrogen–Phosphorus Co-Application on Biomass Allocation and Accumulation in Two-Year-Old *Pinus yunnanensis* Seedlings

**DOI:** 10.3390/biology14091115

**Published:** 2025-08-22

**Authors:** Jianzhen Liao, Yaqi Li, Boning Yang, Chiyu Zhou, Zixing Pan, Lin Chen, Nianhui Cai, Yulan Xu

**Affiliations:** 1 Key Laboratory of Forest Resources Conservation and Utilization in the Southwest Mountains of China, Ministry of Education, Southwest Forestry University, Kunming 650224, China; liaojianzhen@swfu.edu.cn (J.L.); liyaqizzj@163.com (Y.L.); yangboning@swfu.edu.cn (B.Y.); zhouchiyu@swfu.edu.cn (C.Z.); pzixingg@163.com (Z.P.); linchen@swfu.edu.cn (L.C.); cainianhui@swfu.edu.cn (N.C.); 2 Key Laboratory of National Forestry and Grassland Administration on Biodiversity Conservation in Southwest China, Southwest Forestry University, Kunming 650224, China

**Keywords:** *Pinus yunnanensis*, fertilization, biomass, fertilization effect, allometric growth

## Abstract

*Pinus yunnanensis* is a significant native tree species in southwestern China, contributing substantially to the area’s ecological stability and economic growth. However, its growth rate tends to be relatively slow during the seedling stage, and fertilization is crucial to promote seedling growth. This study used two-year-old *P. yunnanensis* seedlings as experimental materials and applied a 3 × 3 factorial design, combining three nitrogen (N) levels (0, 0.4, and 0.8 g·plant^−1^) supplied in the form of urea with three levels of phosphorus (P) (0, 0.8, and 1.6 g·plant^−1^) supplied in the form of superphosphate to form nine treatments, denoted as T1 to T9. This study was carried out in the open-air nursery of Southwest Forestry University, with fertilization beginning in July and observations continuing until December of the same year. Using allometric growth analysis and constructing the fertilizer response regression equation, we investigated the effects of fertilization on biomass accumulation in *P. yunnanensis.* The findings revealed that fertilization significantly increased the biomass allocation ratio to roots but decreased the allocation to needles and aboveground parts (*p* < 0.05). Allometric growth analysis showed that root growth was more rapid than stem and needle growth, and the growth rate of belowground parts exceeded that of aboveground parts. Allometric growth between organs differed among treatments, whereas the allometric growth relationship between aboveground and belowground biomass showed no significant difference across treatments. Moderate N and P fertilizer application promoted biomass accumulation in all organs, with T5 (N: 0.4 g·plant^−1^; P: 0.8 g·plant^−1^) exhibiting the highest biomass accumulation. Based on the comprehensive analysis of optimal N and P fertilizer requirements for biomass accumulation across different organs, the recommended fertilizer application rates are as follows: N 0.5–0.6 g·plant^−1^ and P 0.5–0.9 g·plant^−1^, with an optimal N:P ratio ranging from 1:0.8 to 1:1.8. The results establish a scientific rationale for enhancing fertilization methods in *P. yunnanensis* seedling cultivation, contributing to the slow growth issue during the seedling stage.

## Simple Summary

The *Pinus yunnanensis* is an important tree species in the southwestern region of China. Its seedlings grow slowly, which limits its benefits in terms of ecology and cost-effectiveness Our research tested the effects of nitrogen and phosphorus fertilizers on the growth of seedlings. We designed nine different combinations of nitrogen and phosphorus fertilizers, and measured the biomass of each organ three times after fertilization. The results showed that the fertilizers promoted the accumulation of biomass in each organ and also increased the allocation of biomass to the root. Among the nine treatments we set up, T5 (N: 0.5 g·plant^−1^, P: 0.8 g·plant^−1^) had the best effect on promoting biomass accumulation. Based on the optimal fertilizer requirements for biomass accumulation in each organ, the ideal fertilizer dosage is nitrogen 0.5–0.6 g·plant^−1^ and phosphorus 0.5–0.9 g·plant^−1^, with a nitrogen and phosphorus ratio of 1.0:0.8–1.0:1.8. These findings can help growers optimize their fertilization methods to accelerate the growth rate of seedlings, thereby promoting the healthy growth of forests and the livelihoods of local residents.

## 1. Introduction

Biomass accumulation refers to the total amount of organic matter synthesized by plants through photosynthesis, while biomass allocation describes the distribution of this matter among various plant structures [1,2]. Plants make trade-offs in energy allocation during physiological processes such as growth and reproduction. A common method to assess biomass allocation is to calculate the proportion of biomass in each plant organ relative to the total biomass. This metric may reveal adaptive strategies that plants can adopt in response to resource scarcity or external disturbances [3,4]. Biomass allocation among organs can be influenced by habitat conditions and plant traits [5]. The distribution of leaf, stem, and root biomass has been found to change markedly with latitude, longitude, and altitude and is positively linked to forest age and average yearly precipitation [6]. The Allometric Growth Theory (AGT) describes the disproportionate growth relationships between different tissues of an organism [7]. The theory is used in botany to study the growth relationships between plant parts (e.g., leaf, stem, and root) and to some extent explains the pattern of plant biomass allocation [8]. To sum up, understanding biomass accumulation and allocation patterns and their underlying mechanisms is crucial for elucidating how plants respond to environmental changes and improving plant cultivation and management strategies.

Pine trees are widely distributed throughout the world. *Pinus sylvestris* is the most widely distributed pine species in the northern hemisphere [9]. In the Lake States of the USA, *Pinus banksiana* is an important forest cover type on nutrient-poor sands [10]. Phosphorus (P) deficiency is the most common problem in pine trees [11]. *Pinus halepensis* in the semi-arid southeast of Spain and artificial *Pinus massoniana* forests, which are most common in subtropical and tropical regions of China, are both limited by P [12,13]. *Pinus yunnanensis* is an evergreen coniferous tree species of the genus *Pinus* in the family Pinaceae, primarily distributed in southwestern China [14]. It is both a native and economically important tree species in Yunnan Province [15]. The predominant soil type in southwestern China is ferralsols, which is characterized by a lack of nitrogen (N) and P but rich in potassium (K) [16]. Studies have shown that the seedlings of *P. yunnanensis* experience a growth stagnation period of 3–4 years [17]. This severely compromises reforestation efficiency and constrains the full realization of its ecological and economic value as an important species in Yunnan Province [18].

In practical production, high-quality seedlings are crucial for successful afforestation. Fertilization is an essential management practice in seedling cultivation. Proper fertilization not only promotes plant growth but also enhances soil fertility, increases plant yield, and improves resistance [19,20,21]. In forestry and agriculture, fertilization has become common to supplement essential nutrients and enhance plant growth and productivity [22]. Among these vital nutrients, N and P are particularly important due to their significant roles in numerous physiological activities like photosynthesis, energy transfer, and nucleic acid synthesis [23,24,25]. N is often a limiting factor in terrestrial ecosystems, and its addition can significantly increase plant biomass [26]. However, the response to N fertilization can be constrained by the availability of P in the soil [27]. Currently, the simultaneous application of N and P is acknowledged as a highly effective approach for promoting the growth of seedlings [28]. The response characteristics of plants to N and P differed with different nutrient utilization [29]. A core and undeniable factor driving changes in biomass allocation is nutrient availability [30]. Previous studies have found that fertilization significantly increased the chlorophyll content in *P. yunnanensis* and influenced root morphology to enhance root growth [31,32]. The combined application of N and P significantly influenced biomass accumulation, allocation, and allometric growth relationships in *P. yunnanensis* seedlings after coppicing, promoting the accumulation of biomass and non-structural carbohydrates in the seedlings post-coppicing [33,34]. Although previous studies have investigated the changes in biomass of *P. yunnanensis* seedlings following fertilization, there is a lack of continuous observations on the dynamic changes in biomass allocation. The specific changes in the biomass distribution of *P. yunnanensis* over time after fertilization are still unclear. The biomass allocation of two-year-old *P. yunnanensis* seedlings was dynamically monitored at the end of August, October, and December after fertilization on July 1, to investigate the effects of different N and P fertilizer application rates on seedling growth. Through comprehensive analysis, the research aims to investigate the biomass response of *P. yunnanensis* to combined N and P application, determine the optimal fertilization rate for *P. yunnanensis* growth, and provide theoretical guidance for efficient cultivation of *P. yunnanensis* seedlings. At the same time, it also provides ideas and references for fertilizer research on pine trees in areas with nutrient deficiencies.

## 2. Materials and Methods

### 2.1. Study Area

The experimental site is located in the open-air nursery of Southwest Forestry University in Kunming, Yunnan Province (N 25°04′00″, E 102°45′41″, 1945 m above sea level). The area belongs to North Asia’s subtropical humid plateau monsoon climate, with distinct wet and dry seasons. The average annual precipitation is 942.5 mm, the average temperature is 14.7 °C, the absolute minimum temperature is −9 °C, and the absolute maximum temperature is 32.5 °C. The average annual duration of sunshine is 2445.6 h and the sunshine rate is 56%.

### 2.2. Seedling Cultivation

The experimental seeds were sourced from the half-sibling families of the clonal seed orchard in Midu County, Yunnan Province. Healthy seeds were selected and sown in plug trays at the open-air nursery of Southwest Forestry University. After germination, seedlings were transplanted into plastic pots (16 cm diameter × 20 cm height). The seedlings were cultivated using a mixture of humus and ferralsols, with a ratio of 1:3 (*v*/*v*). In June 2020, we selected well-cultivated two-year-old *P. yunnanensis* seedlings from the open-air nursery of Southwest Forestry University for subsequent fertilization experiments. This choice was based on the fact, as mentioned in the introduction, that *P. yunnanensis* seedlings have a 3–4 years growth stagnation period; our aim in selecting two-year-old seedlings was to promote their growth and shorten the growth stagnation period through fertilization. The seedlings grew evenly, were healthy, and were free of diseases and insect pests. The average seedling height was 8.1 cm, the ground diameter was 9.8 mm, and the needle length was 11.6 cm. Before fertilization, the physical and chemical properties of the seedling culture mixed substrate were tested. These data were merely used as background records. The pH of the mixed substrate, measured in deionized water (1:2.5 *w*/*v*) using a calibrated glass electrode, ranged from 6.0 to 6.2. The contents of total nitrogen (TN) and total phosphorus (TP) of the cultivation mixed substrate were 1.15 g·kg^−1^ and 0.89 g·kg^−1^, respectively. During the seedling cultivation phase, seedlings were irrigated three times per week and weeded at fortnightly intervals.

### 2.3. Experimental Design

Based on previous fertilization experiments conducted by our research group that established preliminary fertilizer application ranges, and aiming to further determine the optimal fertilization rate, we designed the N and P application rates for this study according to Zhang et al.’s method [35]. A two-factor (nitrogen and phosphorus fertilizer), three-level, 3 × 3 regression design was adopted, with three application levels for each fertilizer. The high level was twice the medium level, while the low level was 0 g·plant^−1^. The three levels of N and P combinations resulted in a total of 9 treatments, as shown in Table 1. Urea (with a N content of 46%) and superphosphate (with a P_2_O_5_ content of 12%) were selected as the N and P sources, respectively, owing to their high nutrient concentrations, low cost, and long-standing use as standard fertilizers in local nurseries. Each treatment was set up with three replicates, with each replicate consisting of 60 two-year-old *P. yunnanensis* seedlings, totaling 1080 seedlings across the 9 treatments. This large sample size ensured the randomness of subsequent sampling and effectively reduced experimental error. N and P fertilizers were surface-broadcast uniformly onto the substrate of each pot and immediately irrigated to enhance nutrient uptake. Fertilization commenced on 1 July 2020 and was repeated every 15 days for four applications. Except for the differential fertilizer rates, all subsequent management practices were consistent across treatments.

### 2.4. Sampling and Biomass Measurement

After the fertilization was completed, samples were taken at the end of August, October and December 2020, respectively. For each treatment, 6 plants were randomly selected, and a total of 54 plants were taken from the 9 treatments. Sampling was conducted using the whole-plant excavation method. After collecting the seedlings from the open-air nursery, they were washed with water and divided into roots, stems and needles, and then placed in marked paper bags. To prevent enzymatic carbohydrate reactions, the samples were dried at 105 °C for 30 min to deactivate enzymes, then at 80 °C until achieving a constant weight, and the dry biomass was measured [36,37]. The dry weight of the root, stem, and needle was measured as the biomass, accurate to 0.001 g. The dry weight measurements were used to calculate the biomass allocation ratios (biomass of each component/individual biomass × 100%).

### 2.5. Statistical Data Analysis

The measured data were organized and calculated using Excel 2010. Before statistical analysis, data normality was checked using the Shapiro–Wilk test, and homogeneity of variance was assessed with Levene’s test. One-way analysis of variance (ANOVA) was performed using SPSS 26.0 to analyze biomass accumulation and allocation in *P. yunnanensis* seedlings, with Duncan’s multiple range test for post hoc comparisons (*p* < 0.05). Differences between correlation coefficients were tested using Fisher’s r-to-z transformation and the associated z-test for two independent samples [38,39]. The chart was created using Origin 2021.0. When investigating the response of biomass accumulation to fertilization, it was taken into account that the August data reflected the transition state after fertilization (the last fertilization was completed on August 15), while the growth in December was affected by winter dormancy. Therefore, the October data was prioritized for allometric growth analysis, coefficient of variation, and phenotypic plasticity index calculations, and constructing fertilizer–response regression equations

#### 2.5.1. Coefficient of Variation and Phenotypic Plasticity Index

The phenotypic plasticity index and coefficient of variation were calculated using the following formulas, respectively, to evaluate the degree of change in biomass under different treatments [40,41].

(1) $$\begin{array}{c} \mathrm{PPI}=\frac{Max-Min}{Max} \end{array}$$

(2) $$\begin{array}{c} \mathrm{CV}=\left(\frac{SD}{mean}\right)\times 100\% \end{array}$$

In Equation (1), “*Max*” and “*Min*” represent the average values of the maximum and minimum values of the variable, respectively. In Equation (2), “*SD*” stands for standard deviation, and “*mean*” represents the average value.

#### 2.5.2. Allometric Growth Analysis

The allometric relationship of biomass in *P. yunnanensis* can be represented by the equation *y* = a*x*^b^ [42], which is linearly transformed as:

(3) $$\begin{array}{c} \log y=\mathrm{loga}+\mathrm{blog}x \end{array}$$

In this equation, the variables *y* and *x* denote the biomass accumulation of each component, “a” is defined as the intercept of the trait relationship, and “b” is designated as either the allometric growth index or the equation’s slope. The standardized major axis (SMA) regression analysis was conducted using the Smatr package (V 2.0) in R to calculate the slope parameters of the equation [43] and compare the differences between slopes and between each slope and 1.0 [44]. When there is a significant difference between b and 1.0 (*p* < 0.05), it indicates an allometric growth relationship among different components’ biomass; otherwise, it shows an isometric growth relationship. A typical slope is given if there is no significant difference between the slopes.

#### 2.5.3. Construction of the Fertilizer–Response Regression Equation

Regression analyses were conducted in SPSS 26.0 to quantify the effects of N and P application rates on root, stem, needle, and individual biomass. Three-dimensional response surface plots were then generated in SAS 9.3 to visualize the corresponding biomass–fertilizer relationships [45].

(4) $$\begin{array}{c} Y=a_{0}+a_{1}N+a_{2}P+a_{3}N^{2}+a_{4}P^{2}+a_{5}NP \end{array}$$

where *Y* is the growth trait indicator; a_0_, a_1_, a_2_, a_3_, a_4_, and a_5_ are undetermined coefficients; *N* is the amount of N fertilizer, *P* is the amount of P fertilizer, and *NP* is the interaction term of N and P fertilizers. According to the fitted response-surface equation, the optimal N and P rates and their ratio were determined by partial differentiation.

## 3. Results

### 3.1. Dynamic Responses of Biomass Allocation in P. yunnanensis to N and P Fertilization

**Figure 1:**
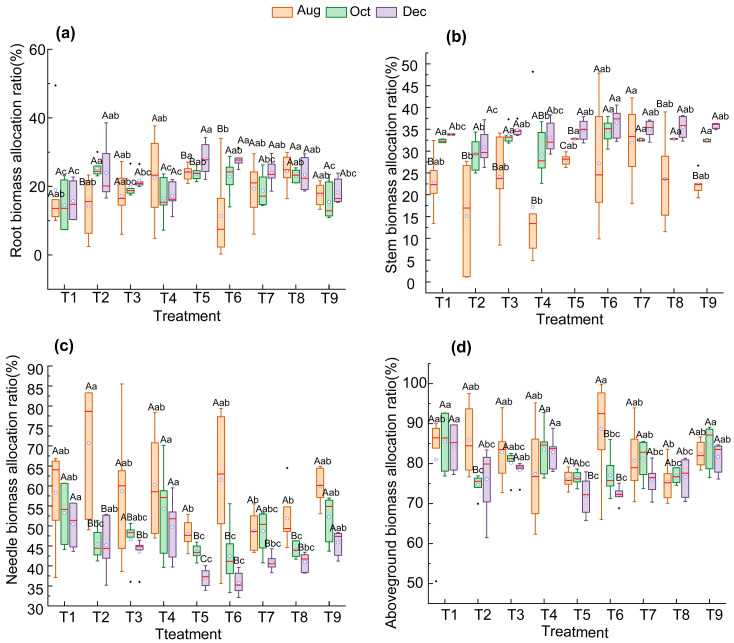
Dynamic changes in biomass allocation ratios of *P. yunnanensis* seedlings under different fertilization treatments: root (**a**), stem (**b**), needle (**c**), and aboveground (**d**). Different capital letters indicate significant differences between treatments at different times, while different lowercase letters indicate significant differences between treatments at the same time. In the boxplot, the box represents the middle 50% of the data, with the bottom edge indicating the first quartile (Q1), the top edge indicating the third quartile (Q3), and the line inside the box representing the median. The height of the box corresponds to the interquartile range (IQR), which is the difference between Q3 and Q1. The error bars extend to the upper and lower bounds within 1.5 × IQR from Q1 and Q3, respectively, while any points beyond these bounds are considered outliers and are marked with a small circle. White circles represent the mean values of the data (*n* = 6).

In order to investigate the dynamic characteristics of biomass allocation after fertilization, the biomass allocation of each organ in August, October, and November was analyzed dynamically (Figure 1). Biomass distribution patterns of various organs in *P. yunnanensis* are: aboveground > belowground; needle > stem > root. Compared with no fertilization (T1), the biomass allocation of *P. yunnanensis* changed after fertilization. The needle biomass allocation in multiple fertilization treatments was lower than that in T1 (Figure 1c). Among them, all treatments showed no significant difference from T1 in August, while T5 and T6 in October and T3, T5, T6, T7, and T8 in December were significantly lower than T1 (*p* < 0.05). Stem biomass allocation is less affected by fertilization (Figure 1b). T2, T5, T6 and T8 all showed significantly greater root biomass allocation than T1 in October and December (*p* < 0.05) (Figure 1a), while their aboveground biomass allocation was significantly lower than that of T1 (*p* < 0.05) (Figure 1d). After fertilization, the overall trend was an increase in root biomass allocation, but there was a decrease in needle and aboveground biomass allocation, with the effects of T5 and T6 (N and P combined application) being particularly significant. This is an adjustment strategy of seedlings in response to changes in the external environment.

In terms of time, with seedling development, there are differences in the changes in biomass allocation over time between treatments. From August to December, root biomass allocation showed a significant increase in T2 and T5 (*p* < 0.05), while no significant differences were observed in the remaining treatments (Figure 1a); stem biomass allocation showed a significant increase in all treatments except T7 (*p* < 0.05) (Figure 1b); conversely, needle biomass showed a significant decrease in T2, T3, T5, T6, T7, and T8 (p < 0.05) (Figure 1c); aboveground biomass showed a significant decrease in T2, T5, and T6 (*p* < 0.05) (Figure 1d). It is worth noting that as the seedlings develop, T5 (N: 0.4 g·plant^−1^; P: 0.8 g·plant^−1^) exhibited a significant increase in root and stem biomass allocation (*p* < 0.05), while needle and aboveground biomass allocation showed a significant decrease (*p* < 0.05). Overall, the allocation of root and aboveground biomass is less affected by time, with relatively stable allocation at different times (*p* < 0.05). Stem biomass allocation increases with time, while needle biomass allocation decreases with time (*p* < 0.05).

### 3.2. Analysis of Allometric Growth of Root, Stem, Needle, and Aboveground Biomass

**Figure 2:**
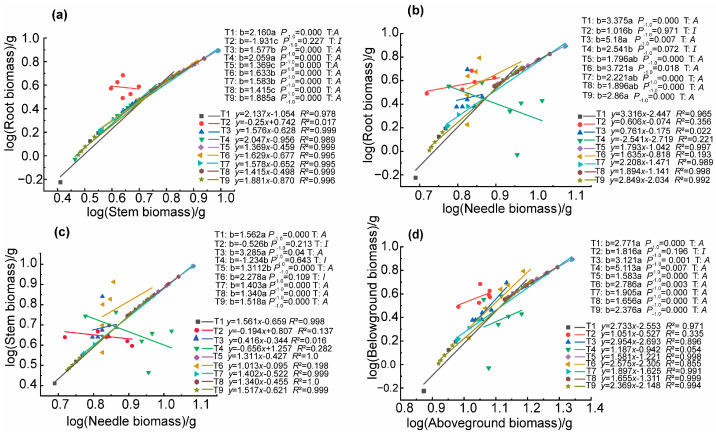
The allometric growth relationships between organ biomass in October, root and stem (**a**), root and needle (**b**), stem and needle (**c**), and belowground and aboveground (**d**), were fitted for the different fertilizer treatments. *P_−1.0_* indicates the significance between the slope and the theoretical value of 1.0. *T* denotes the type of growth relationship, *A* denotes allometric growth, and *I* denotes isotropic growth (*n* = 6).

In October, an allometric growth analysis was conducted on the biomass accumulation of various organs of seedlings under different fertilization treatments using SMA analysis. The results are shown in Figure 2. The root–stem (Figure 2a) and the belowground–aboveground parts (Figure 2b) showed isometric growth in treatment T2, while they showed allometric growth in the remaining treatments; that is, the growth rate of the root was faster than that of the stem. The growth rate of the belowground parts was greater than that of the aboveground parts. The root–needle (Figure 2b) showed allometric growth except in treatments T2 and T4. The stem–needle (Figure 2c) showed allometric growth in T1, T3, T5, T7, T8, and T9. Overall, the growth rates of various organs were as follows: root > stem > needle. At the same time, we found differences in the allometric growth indices of root–stem, root–needle, and stem–needle under different fertilization treatments. However, the allometric growth index of belowground and aboveground parts showed no significant difference. This indicates that fertilization changed the growth trajectories of root–stem, root–needle, and stem–needle but did not change that of the belowground–aboveground parts.

### 3.3. Analyzing the Coefficient of Variation and Phenotypic Plasticity Index

**Figure 3:**
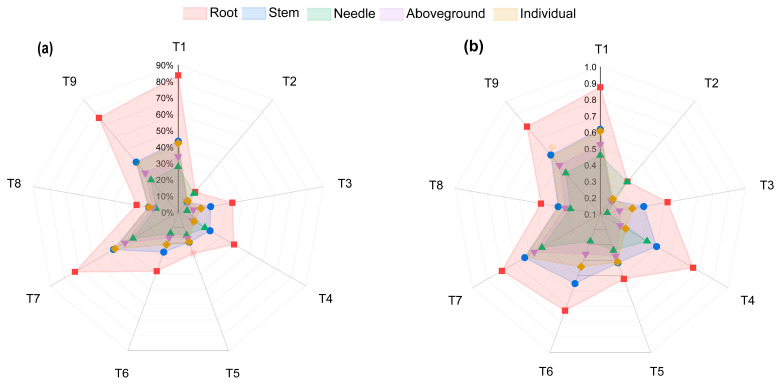
Coefficient of variation (**a**) and phenotypic plasticity index (**b**) of the root, stem, needle, aboveground, and individual biomass of *P. yunnanensis* seedlings under nine fertilization treatments.

The coefficient of variation (CV) and phenotypic plasticity index (PPI) of each index showed the following order: root > stem > individual plant > aboveground part > needle (Figure 3a,b). The CV and PPI range of each index were as follows: root: 16.17–83.64%, 0.35–0.87; stem: 8.36–45.67%, 0.21–0.63; needle: 5.48–31.78%, 0.14–0.51; aboveground: 8.38–37.48%, 0.20–0.57; individual: 9.06–44.72%, 0.22–0.63. The CV and PPI of the root were significantly elevated compared to other organs and reached the maximum value (83.64%, 0.87) in the unfertilized treatment (T1). Notably, the CV and PPI of the stem, needle, single plant, and aboveground part of high-N treatment (T7) were higher than those of other fertilization treatments except T1.

### 3.4. Construction of the Effect Equation of Nitrogen and Phosphorus Addition

By analyzing the accumulation of biomass of *P. yunnanensis* under different fertilization treatments in October, we found that the biomass of root, stem, needle and individual in the T5 treatment was the highest (Table 2). Based on the determined biomass accumulation under fertilization treatment, regression equations were established to explore the relationship between the biomass accumulation of each organ and the dosage of N and P fertilizers. Based on the correlation coefficient, significance level and residual standard deviation of the regression equation, the optimal regression equation was finally selected (Table 3).

**Figure 4:**
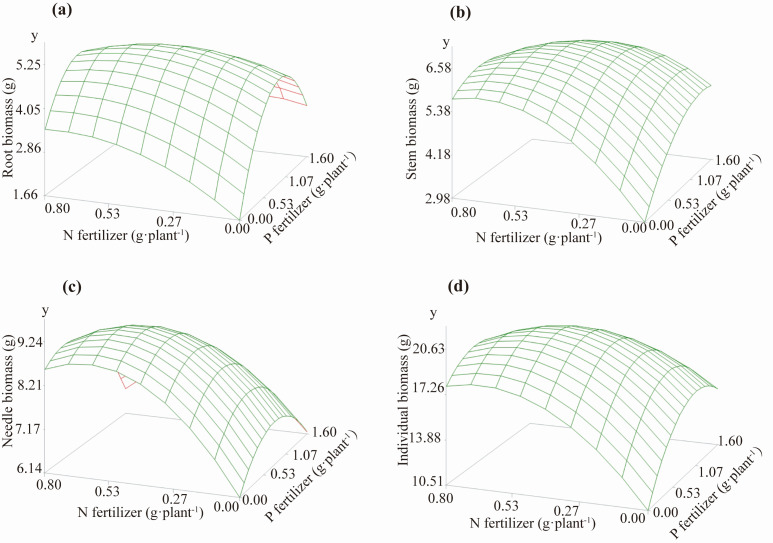
Fertilizer response surface plots for biomass of *P. yunnanensis* seedlings: root (**a**), stem (**b**), needle (**c**), and individual (**d**).

Based on the regression equations in Table 3, a 3D surface plot of fertilizer effects was created using SAS 9.3 (Figure 4). Figure 4 shows that the effect of fertilizer on biomass accumulation follows a quadratic parabolic pattern, which means that both the biomass of each organ and the biomass of individual plants have a peak. Before reaching the peak, all growth indicators increase with increasing N and P application rates. After the peak, all growth indicators decrease with increasing N and P application rates. This indicates that different organs of *P. yunnanensis* have similar nutritional requirements for N and P. Appropriate fertilization can promote biomass accumulation in all organs of *P. yunnanensis*. However, exceeding the optimal dose not only fails to achieve the best results but also leads to fertilizer waste.

By taking partial derivatives of the regression equations established in Table 3, we determined the optimal N and P fertilization rates for biomass accumulation in each organ of 2-year-old *P. yunnanensis* (Table 4). As shown in Table 4, the most suitable N and P application rates and ratios vary slightly among different organs of *P. yunnanensis* seedlings. Overall, the N application rate ranges from 0.5 to 0.6 g·plant^−1^, and the P application rate ranges from 0.5 to 0.9 g·plant^−1^, with the optimal N:P ratio being 1.0:0.8–1.0:1.8. Among the fertilization treatments designed in this study, the one closest to the above results was T5 (N: 0.4 g·plant^−1^; P: 0.8 g·plant^−1^).

### 3.5. Impact of Fertilization on the Correlation of Biomass Among Various Organs of P. ynnanensis Seedlings

To investigate whether fertilization alters the correlations among biomass of various organs in *P. yunnanensis* seedlings, we categorized the fertilization treatments as follows: unfertilized (T1), N-fertilized (T4, T7), P-fertilized (T2, T3), and N + P (T5, T6, T8, T9). Using Fisher’s z-transformation and two-tailed z-tests, we compared the Pearson correlation coefficients between organ biomasses under the unfertilized treatment and the three fertilization treatments one by one (α = 0.05). The results showed that fertilization did not alter the direction of the biomass correlation coefficients between organs but did change their magnitude. When N was applied alone (Table 5), the biomass correlation coefficients between roots and other organs increased, but the differences were not significant. The most significant change was observed in the stem–needle correlation coefficient, which decreased from r = 0.909 to 0.736, but no significant difference was detected; similar trends were observed when P was applied alone (Table 6). In contrast, combined N and P application increased the root–aboveground biomass correlation coefficient from 0.668 to 0.887 and the root–individual biomass correlation coefficient from 0.832 to 0.948, both of which were significantly different (*p* < 0.05) (Table 7). The remaining differences were not significant (*p* > 0.05). Thus, the synergistic supply of N and P strengthened the coupling between the root system and aboveground and individual biomass, which has positive implications for maintaining overall growth balance. However, the application of a single fertilizer did not significantly alter the relationships between organs.

## 4. Discussion

Fertilization is a management measure in the cultivation of seedlings; reasonable fertilization can promote the growth of seedlings and improve their quality [46]. The root system serves as the primary plant organ for direct nutrient uptake from soil to facilitate plant growth [47]. The introduction of nutrient elements significantly affects root biomass [48,49]. Research on nitrogen application in rice demonstrates that total root length, root surface area, and root biomass all increase with elevated nitrogen levels [50]. Studies indicate that plants typically modify their root architecture in response to varying external nutrient conditions, with nitrogen deficiency stimulating root elongation [51]. Combined N and P application promotes increases in both root length and surface area [52]. Plants achieve environmental adaptation and growth promotion by optimizing biomass allocation to support metabolic and physiological processes [53]**.** Under certain environmental conditions, seedlings will sacrifice the biomass allocation of leaf and increase the biomass of root [54]. In this study, compared to the non-fertilized treatment (T1), fertilization increased the biomass allocation ratio of the root. Conversely, it decreased the biomass allocation ratio of the needle and aboveground. In October, the root biomass allocation of T5 (medium N and medium P) increased significantly, with a biomass allocation ratio 1.63 times that of T1. indicating that the seedlings increased the input to the root system after fertilization. At this stage, the root and individual biomass accumulation in T5 were the highest and significantly greater than in the unfertilized T1 (*p* < 0.01). This might be due to the increase in nutrient content in the soil after fertilization, making the function of the root system in absorbing water and nutrients even more crucial. The root system requires more biomass to expand the absorption area and improve the absorption efficiency, thereby making more effective use of these additional nutrients [55,56]. A recent meta-analysis indicated that nitrogen application altered the distribution pattern of biomass: a higher proportion of biomass was allocated to aboveground, such as leaf and stem, while a lower proportion was allocated to root [26]. In our study, combined N and P application (T5) increased root biomass allocation, which may be attributed to the combined limitation of N and P on *P. yunnanensis* seedlings before fertilization, as well as differences resulting from the combined use of P fertilizer. In Tang et al.’s study [57], N can enhance the activity of key enzymes and proteins involved in phosphorus absorption, promoting root growth to increase P uptake; in low-P environments, N can catalyze the recycling of P, while P helps plants absorb N. The availability of N and P in the soil limits plant growth, thereby affecting the C flux of the ecosystem [58,59]. The study by Zhai et al. [60] demonstrated that N and P additions increased soil N and P availability, which further improved leaf stoichiometric traits and subsequently altered biomass allocation patterns. In the later stage, we can also explore the effects of fertilization on the stoichiometric ratios of *P. yunnanensis* from the perspective of stoichiometry, and analyze the relationship between stoichiometric ratios and biomass allocation. In addition, in our study, aboveground biomass allocation exceeded belowground, indicating that resources are preferentially directed to the aerial organs [61]. As the seedlings developed, root biomass allocation remained relatively stable, whereas stem allocation increased and needle allocation decreased. This pattern likely reflects a coordinated strategy among *P. yunnanensis* seedling organs, prioritizing investment in the stem, because the development of needles depends on the positions provided by the stem. This provides the basis for needle to obtain energy through photosynthesis in the later stages.

Allometric relationships are frequently employed to explore the mechanisms of biomass allocation across diverse organ systems [62]. In small and short-lived herbaceous plants, the biomass of reproductive and vegetative organs often exhibits a linear relationship. In contrast, in long-lived, large plants, the relationship between the biomass of these organs is characterized by allometric growth, with a scaling exponent typically less than 1.0 [63]. Enquist and Niklas [42], through a comprehensive analysis of biomass data from seed plants worldwide, concluded that leaf biomass follows a 3/4 scaling relationship with stem and root biomass, while root biomass scales isometrically with stem biomass. In our results, the ratio of needle biomass to root and stem biomass exceeds 3/4. Meanwhile, root and stem biomass do not grow isometrically; instead, roots grow faster. This may be attributed to the species itself and the climatic environment where the plants are located. The results showed that under different fertilization treatments, allometric relationships were observed in most cases for biomass accumulation in root, stem, needle, and aboveground parts. These allometric relationships reflect adaptive strategies for optimizing resource utilization [61]. In most treatments, roots exhibit a higher growth rate compared to stems and needles, with belowground biomass accumulation surpassing that of aboveground parts. This indicates that roots are closely related to nutrient absorption and utilization, and that they prioritize the acquisition and utilization of resources. [64]. We found that in T2 of this experiment, isometric relationships existed between the biomass of various organs and between aboveground and belowground biomass. This result has also been reported in previous studies [65,66]. This may be because the application of 0.8 g·plant^−1^ of P fertilizer created a relatively stable environment, eliminating the need to prioritize investment in any specific organ and maintaining balanced growth across all organs.

Both the CV and PPI can reflect the stability of measured parameters, with higher CV values indicating lower stability and vice versa [67]. In this study, the CV and PPI results exhibited consistent patterns. The needle showed the lowest CV values, while the root demonstrated the highest CV values, indicating the most stable biomass accumulation in the needle and the least stable accumulation in the root. This phenomenon arises because root exhibit heightened environmental responsiveness as the primary organs for resource acquisition from the soil. The unfertilized control treatment (T1) showed the maximum CV (83.64%) and PPI (0.87) in the root, suggesting poor root stability under nutrient-deficient conditions. Fertilization can enhance root stability, which aligns with previous studies demonstrating that nutrient supplementation reduces phenotypic variability in root [68]. However, stable root biomass accumulation may reduce the ability to rapidly adjust morphology (such as root length or branching changes) when nutrient supply suddenly decreases, which may weaken the ability to adapt to environmental disturbances [69]. From an ecological perspective, the stability of root biomass accumulation is expected to improve nutrient absorption and distribution efficiency, enabling nutrients to be transported more effectively to aboveground organs, thereby promoting the sustained growth of seedlings and reducing the risk of mortality in the early stages, which is crucial for forest regeneration in nutrient-deficient areas [70]. N is essential for plant protein synthesis and photosynthesis, but excessive N may induce the accumulation of toxic intermediates (such as ammonium), disrupting metabolic homeostasis [27,71]. T7 had higher CV and PPI values. Under high N conditions, T8 showed reduced CV and PPI values after applying moderate amounts of P fertilizer, while T9 maintained high CV and PPI values even after applying high amounts of P fertilizer. This indicates that a reasonable N-P application can reduce CV and PPI values, and appropriate phosphorus fertilizer addition promotes N absorption while preventing excessive ammonium accumulation.

N and P, as essential mineral elements for plants, are also the most common limiting factors in plant growth [72]. Fertilization can affect the nutrient content of plants [73], leading to alterations in the accumulation and allocation of biomass in seedlings [74]. N and P fertilization significantly enhanced the biomass accumulation of two-year-old *P. yunnanensis*, alleviating the issue of slow growth. The response of *P. yunnanensis* biomass accumulation to N and P application follows a quadratic parabolic surface. Plants have a specific tolerance limit for any factor, and when this factor approaches or exceeds the plant’s tolerance range, it restricts the growth of plants [75]. Appropriate fertilization ensures the expected growth of trees [76]. The response surface plots of fertilizer effects (Figure 4) and the measured biomass (Table 2) show that all fertilization treatments in this study increased the biomass accumulation of the seedlings, indicating that our experiment did not involve excessive fertilization. However, whether it was the application of N alone, P alone, or the combined application of N and P, the biomass of each organ first increased and then decreased with the increase in fertilizer application rates, indicating the existence of an optimal fertilization treatment. Exceeding the optimal N fertilization may lead to an imbalance in the carbon-nitrogen ratio within plants, inhibiting the allocation of photosynthetic products and thereby affecting the growth of root and aboveground parts [77]. High concentrations of fertilizer modify soil osmotic pressure, impairing the nutrient absorption capability of plant root [78]. Research indicates that N and P act synergistically in plant metabolism [79], and appropriate N-P coapplication can enhance nutrient use efficiency. Numerous studies have demonstrated that fertilization promotes seedling growth and enhances biomass accumulation [33,80,81,82]. This study found that under a fixed N application rate, increasing P fertilizer led to an initial rise followed by a decline in the biomass of organs and the whole plant. The maximum biomass accumulation across various organs was achieved under T5 (N: 0.4 g·plant^−1^; P: 0.8 g·plant^−1^), indicating that the combined application of moderate levels of N and P fertilizers is most conducive to biomass accumulation in two-year-old *P. yunnanensis.* In October, based on our actual measurement data, the biomass of root, stem, and needle of T5 increased by 3.2 g, 3.2 g, and 3.2 g, respectively, compared with that of T1 without fertilization treatment, and the individual biomass increased by 9.6 g. The biomass of each organ in group T5 was extremely significantly higher than that in the control group T1 (*p* < 0.01). The biomass accumulation of all organs peaked under the T5. We also found that the suitable N and P fertilizer application rates for two-year-old *P. yunnanensis* seedlings ranged from 0.5 to 0.6 g·plant^−1^ for N and 0.5–0.9 g·plant^−1^ for P, with an appropriate N-P Fertilizer ratio of 1.0: 0.8–1.0: 1.8. Among the treatments in this study, T5 was closest to the above-mentioned recommended N and P application rates and ratios. Table 4 presents the optimal N and P application rates and ratios determined in this study, which can serve as a reference for the practical cultivation of *P. yunnanensis* seedlings. The application rates required to maximize biomass differ slightly among organs and can be selected according to specific needs. For example, using the fertilizer rate that maximizes individual biomass (N: 0.5 g·plant^−1^, P: 0.8 g·plant^−1^) and substituting these values into the regression equations in Table 3, individual biomass yield is predicted to increase by 96% compared with the unfertilized control.

There is a strong synergistic relationship between the sizes of the aboveground and underground parts of plants [83,84]. Plants absorb nutrients from the soil and transport them to the aboveground parts; after acquiring these nutrients, the aboveground parts exhibit enhanced photosynthesis, synthesize more energy, and transport it to the root, which in turn promotes root growth [85]. Our results showed that, under the N-P combined treatment, the correlation coefficients between root biomass and aboveground biomass, as well as between root biomass and individual biomass, differed significantly from those of the unfertilized T1. Indicated N-P combined application compensates for the metabolic limitations of single nutrient application, driving the dynamic balance of resource allocation between the “underground and aboveground” parts of *P. yunnanensis* toward a more efficient direction. This enhances the growth synergy among organs and provides a more optimal metabolic basis for the accumulation of total plant biomass.

## 5. Limitations and Future Directions

Although this study offers valuable insights into the effects of fertilization on biomass accumulation and allocation in *P. yunnanensis* seedlings, several limitations must be acknowledged. First, the experimental design focused solely on N–P interactions, whereas field conditions involve more complex nutrient dynamics; interactions involving other soil elements such as K, Ca, and Mg were not considered. Future work should therefore implement multi-nutrient factorial trials (e.g., N–P–K–Mg) to capture these broader interactions. Second, all seedlings originated from a single seed source; while this reduced genetic variance in the present experiment, it may not reflect the diversity found in wild populations across different elevations. Subsequent studies should employ seedlings from multiple provenances and increase the experimental period to refine universally applicable fertilization recommendations. Finally, this investigation remained at a fundamental level and did not explore the molecular mechanisms underlying fertilization-induced changes in biomass. Subsequent research should comprehensively explore these aspects, such as gene expression, signal transduction, metabolic processes, and hormone regulation, to elucidate the molecular mechanisms involved and provide theoretical support for precision seedling cultivation.

## 6. Conclusions

This study investigated the effects of nitrogen and phosphorus fertilizers on the biomass distribution and accumulation of *P. yunnanensis* seedlings. After fertilization, the allocation of root biomass increased, accompanied by a decrease in the allocation of resources to needle and aboveground (*p* < 0.05). Throughout the observation period, as the seedlings grew, the biomass allocation between the root and stem remained stable, with an increase in stem biomass distribution and a decrease in needle biomass distribution (*p* < 0.05). Allometric growth analysis revealed that the growth rate of the root was higher than that of the stem and needle (root > stem > needle), and the growth rate of belowground parts was faster than that of aboveground parts. Notably, fertilization modified organ-specific growth trajectories (root–stem, root–needle, stem–needle) but preserved the proportional balance between belowground and aboveground biomass accumulation. Fertilization reduces the CV and PPI of biomass accumulation in different organs of *P. yunnanensis*. This indicates that fertilization enhances the stability of individual organs, yet it also reduces the plant’s ability to withstand sudden environmental changes. The response of biomass accumulation to N and P application followed a quadratic parabolic trend, highlighting the importance of optimal fertilization levels. Medium N and medium P combination (T5) achieved the highest biomass accumulation, with root, stem, needle, and individual biomass increasing by 3.2 g, 3.2 g, 3.2 g, and 9.6 g, respectively, relative to the control. Based on the constructed regression equation, we determined the optimal range of fertilizer application amounts as follows: N: 0.5–0.6 g·plant^−1^, P: 0.5–0.9 g·plant^−1^, and the optimal N:P ratio is 1.0: 0.8–1.0: 1.8 (integrated with the optimal N and P fertilizer dosages and ratios for all organs). Combined N and P fertilization and the correlation coefficients between root and aboveground biomass, as well as between root and individual biomass, were significantly increased compared to the unfertilized T1 (*p* < 0.05). In contrast, neither N nor P application alone showed significant effects. These results demonstrate that N-P coapplication enhances the growth coordination between roots and the aboveground part. These findings provide a theoretical and practical basis for fertilization management in *P. yunnanensis* seedling cultivation, contributing to solutions for slow growth in the seedling phase. The results of this study provide certain references for the operators of commercial *P. yunnanensis* and the seedling cultivation workers in afforestation in the southwest region.

## Figures and Tables

**Table 1:** The mass of nitrogen and phosphorus fertilizers applied in a single application.

Treatment	N (g·Plant^−1^)	P (g·Plant^−1^)
N1P1 (T1)	0	0
N1P2 (T2)	0	0.8
N1P3 (T3)	0	1.6
N2P1 (T4)	0.4	0
N2P2 (T5)	0.4	0.8
N2P3 (T6)	0.4	1.6
N3P1 (T7)	0.8	0
N3P2 (T8)	0.8	0.8
N3P3 (T9)	0.8	1.6

**Table 2:** The accumulation of biomass of *P. yunnanensis* after fertilization in October.

Treatment	N (g·Plant^−1^)	P (g·Plant^−1^)	Root Biomass (g)	Stem Biomass (g)	Needle Biomass (g)	Individual Biomass (g)
T1	0	0	2.14 ± 1.79 d	4.12 ± 1.79 d	6.44 ± 1.79 d	12.69 ± 5.36 c
T2	0	0.8	3.85 ± 0.62 abcd	4.42 ± 0.37 cd	6.93 ± 1.04 cd	15.20 ± 1.38 c
T3	0	1.6	2.96 ± 1.00 cd	4.94 ± 1.00 cd	6.74 ± 0.37 cd	14.64 ± 2.06 c
T4	0.4	0	2.63 ± 1.04 cd	4.61 ± 1.04 cd	8.46 ± 1.59 abc	15.69 ± 1.76 bc
T5	0.4	0.8	5.35 ± 1.44 a	7.33 ± 1.44 a	9.65 ± 1.44 a	22.33 ± 4.32 a
T6	0.4	1.6	4.16 ± 1.59 abc	6.14 ± 1.59 abc	7.26 ± 1.00 cd	17.56 ± 3.72 abc
T7	0.8	0	3.33 ± 2.42 bcd	5.31 ± 2.42 bcd	7.63 ± 2.42 bcd	16.27 ± 7.27 bc
T8	0.8	0.8	4.93 ± 1.27 ab	6.91 ± 1.27 ab	9.23 ± 1.27 ab	21.07 ± 3.81 ab
T9	0.8	1.6	2.24 ± 1.69 cd	4.22 ± 1.68 cd	6.54 ± 1.69 cd	13.00 ± 5.07 c

Note: Different letters indicate significant differences, and the same letters mean insignificant differences (*p* < 0.05), Duncan test.

**Table 3:** Regression equation of growth characteristics and N and P fertilizer dosage of *P. yunnanensis*.

Index	Regression Equation	*R* ^2^	*F*	*P*
root biomass	*Y* = 1.663 + 4.896*N* + 6.102*P* − 3.289*N*^2^ − 3.235*P*^2^ − 2.125*NP*	0.370	3.521	0.013
stem biomass	*Y* = 2.982 + 8.365*N* + 3.147*P* − 6.155*N*^2^ − 1.118*P*^2^ − 2.153*NP*	0.336	3.030	0.025
needle biomass	*Y* = 6.136 + 8.641*N* + 2.518*P* − 6.986*N*^2^ − 1.552*P*^2^ − 1.462*NP*	0.398	3.973	0.007
individual biomass	*Y* = 10.508 + 23.199*N* + 10.260*P* − 17.486*N*^2^ − 4.694*P*^2^ − 5.769*NP*	0.359	3.355	0.016

Note: *N* represents the amount of nitrogen fertilizer used, and *P* represents the amount of phosphorus fertilizer used, *Y* represents biomass accumulation.

**Table 4:** The optimal fertilizer amount for two-year-old *P. yunnanensis* seedlings to achieve peak biomass yield.

Index	Suitable Amount of Fertilizer				Optimum N and P Ratio
	N (g·Plant^−1^)	95% CI	P (g·Plant^−1^)	95% CI	
root biomass	0.5	[0.00, 0.80]	0.8	[0.22, 1.02]	1.0:1.6
stem biomass	0.5	[0.32, 0.80]	0.9	[0.00, 1.60]	1.0:1.8
needle biomass	0.6	[0.42, 0.80]	0.5	[0.00, 0.86]	1.0:0.8
individual biomass	0.5	[0.40, 0.80]	0.8	[0.00, 1.11]	1.0:1.6
appropriate range	0.5–0.6	\	0.5–0.9	\	1.0:0.8–1.0–1.8

**Table 5:** The influence of a single application of N fertilizer on the correlation coefficients of biomass of various organs of *P. yunnanensis* seedlings.

Trait Index	r1 (Unfertilized)	r2 (N-Fertilized)	z1	z2	z	*p*	Significance
Root–Stem	0.680 (0.002)	0.741 (<0.001)	0.8291	0.9528	−0.397	0.691	ns
Root–Needle	0.624 (0.006)	0.691 (<0.001)	0.7314	0.8497	−0.38	0.704	ns
Root–Aboveground	0.668 (0.002)	0.769 (<0.001)	0.807	1.0175	−0.676	0.499	ns
Root–Individual	0.832 (<0.001)	0.876 (<0.001)	1.1945	1.3585	−0.527	0.598	ns
Stem–Needle	0.909 (<0.001)	0.736 (<0.001)	1.5215	0.9415	1.862	0.063	ns
Stem–Aboveground	0.978 (<0.001)	0.934 (<0.001)	2.2495	1.689	1.799	0.072	ns
Stem–Individual	0.956 (<0.001)	0.924 (<0.001)	1.897	1.616	0.902	0.367	ns
Needle–Aboveground	0.976 (<0.001)	0.930 (<0.001)	2.2055	1.6585	1.756	0.079	ns
Needle–Individual	0.963 (<0.001)	0.906 (<0.001)	1.9855	1.5045	1.544	0.123	ns

Note: r: Pearson correlation coefficient; z1, z2: Fisher-transformed z-values for control and treated groups; z: Fisher’s z-test statistic for correlation difference, |z| > 1.960, then *p* < 0.05, indicating a significant difference in the correlation coefficients; ns: indicates no significant difference between correlation coefficients (*p* > 0.05). The same below.

**Table 6:** The influence of a single application of P fertilizer on the correlation coefficients of biomass of various organs of *P. yunnanensis* seedlings.

Trait Index	r1 (Unfertilized)	r2 (P-Fertilized)	z1	z2	z	*p*	Significance
Root–Stem	0.680 (0.002)	0.816 (<0.001)	0.8291	1.145	−1.014	0.31	ns
Root–Needle	0.624 (0.006)	0.705 (<0.001)	0.7314	0.877	−0.467	0.64	ns
Root–Aboveground	0.668 (0.002)	0.799 (<0.001)	0.807	1.095	−0.925	0.355	ns
Root–Individual	0.832 (<0.001)	0.903 (<0.001)	1.1945	1.49	−0.949	0.343	ns
Stem–Needle	0.909 (<0.001)	0.827 (<0.001)	1.5215	1.1875	1.072	0.284	ns
Stem–Aboveground	0.978 (<0.001)	0.961 (<0.001)	2.2495	1.977	0.875	0.382	ns
Stem–Individual	0.956 (<0.001)	0.958 (<0.001)	1.897	1.8855	0.037	0.971	ns
Needle–Aboveground	0.976 (<0.001)	0.950 (<0.001)	2.2055	1.8315	1.201	0.23	ns
Needle–Individual	0.963 (<0.001)	0.913 (<0.001)	1.9855	1.5045	1.544	0.123	ns

Note: ns: indicates no significant difference between correlation coefficients (*p* > 0.05).

**Table 7:** The influence of N and P combined application on the correlation coefficients of biomass of various organs of *P. yunnanensis* seedlings.

Trait index	r1 (Unfertilized)	r2 (N + P)	z1	z2	z	*p*	Significance
Root–Stem	0.680 (0.002)	0.858 (<0.001)	0.8291	1.2855	−1.602	0.109	ns
Root–Needle	0.624 (0.006)	0.847 (<0.001)	0.7314	1.2465	−1.808	0.071	ns
Root–Aboveground	0.668 (0.002)	0.887 (<0.001)	0.807	1.4105	−2.118	0.034	*
Root–Individual	0.832 (<0.001)	0.948 (<0.001)	1.1945	1.8059	−2.146	0.032	*
Stem–Needle	0.909 (<0.001)	0.849 (<0.001)	1.5215	1.257	0.928	0.353	ns
Stem–Aboveground	0.978 (<0.001)	0.973 (<0.001)	2.2494	2.092	0.552	0.581	ns
Stem–Individual	0.956 (<0.001)	0.959 (<0.001)	1.897	1.9335	−0.128	0.898	ns
Needle–Aboveground	0.976 (<0.001)	0.948 (<0.001)	2.2055	1.8059	1.403	0.161	ns
Needle–Individual	0.963 (<0.001)	0.939 (<0.001)	1.9856	1.7298	0.898	0.369	ns

Note: * indicates a significant difference between correlation coefficients (*p* < 0.05); ns: indicates no significant difference between correlation coefficients (*p* > 0.05).

## Data Availability

The original contributions presented in this study are included in the article. Further inquiries can be directed to the corresponding authors.

## Abbreviations

The following abbreviations are used in this manuscript:

AGT	Allometric Growth Theory
SMA	Standardized Major Axis
CV	Coefficient of variation
PPI	Phenotypic plasticity index
